# Validation of High-Resolution Tractography Against *In Vivo* Tracing in the Macaque Visual Cortex

**DOI:** 10.1093/cercor/bhu326

**Published:** 2015-03-18

**Authors:** Hojjatollah Azadbakht, Laura M. Parkes, Hamied A. Haroon, Mark Augath, Nikos K. Logothetis, Alex de Crespigny, Helen E. D'Arceuil, Geoffrey J. M. Parker

**Affiliations:** 1 Centre for Imaging Sciences, Faculty of Medical and Human Sciences, University of Manchester, Manchester Academic Health Sciences Centre, Manchester, UK; 2 Max Planck Institute for Biological Cybernetics, Tübingen, Germany; 3 Athinoula A. Martinos Center, Massachusetts General Hospital, Charlestown, MA, USA

**Keywords:** diffusion imaging, macaque, tractography, validation, visual cortex

## Abstract

Diffusion magnetic resonance imaging (MRI) allows for the noninvasive in vivo examination of anatomical connections in the human brain, which has an important role in understanding brain function. Validation of this technique is vital, but has proved difficult due to the lack of an adequate gold standard. In this work, the macaque visual system was used as a model as an extensive body of literature of in vivo and postmortem tracer studies has established a detailed understanding of the underlying connections. We performed probabilistic tractography on high angular resolution diffusion imaging data of 2 ex vivo, in vitro macaque brains. Comparisons were made between identified connections at different thresholds of probabilistic connection “strength,” and with various tracking optimization strategies previously proposed in the literature, and known connections from the detailed visual system wiring map described by Felleman and Van Essen (1991; FVE91). On average, 74% of connections that were identified by FVE91 were reproduced by performing the most successfully optimized probabilistic diffusion MRI tractography. Further comparison with the results of a more recent tracer study ( Markov et al. 2012) suggests that the fidelity of tractography in estimating the presence or absence of interareal connections may be greater than this.

## Introduction

Magnetic resonance diffusion images allow in vivo estimation of cerebral anatomical connectivity patterns using techniques such as tractography. However, there is still a need for an adequate gold standard against which such techniques could be validated (Hubbard and Parker 2009; Johansen-Berg and Rushworth 2009).

One approach to validation is to use computer-generated software phantoms (Gossl et al. 2002; Tournier et al. 2002; Lazar and Alexander 2003; Alexander 2005; Leemans et al. 2005; Watanabe et al. 2006; Descoteaux et al. 2007; Iturria-Medina et al. 2007; Sakaie and Lowe 2007) or physical phantoms (Basser et al. 1994; van Doorn et al. 1996; Van Donkelaar et al. 1999; von dem Hagen and Henkelman 2002; Lin et al. 2003; Fieremans et al. 2005; Perrin et al. 2005; Yanasak and Allison 2006; Hubbard et al. 2015). These phantoms are relatively easy to define and manipulate by the user, but may grossly over-approximate the in vivo situation that is being simulated, as the complexities of white matter structures are difficult to reproduce. A priori knowledge of human neuroanatomy (Tournier et al. 2002; Abe et al. 2004; Campbell et al. 2005; Savadjiev et al. 2006; Behrens et al. 2007) and circumstantial evidence from functional imaging studies (Guye et al. 2003; Toosy et al. 2004; Powell et al. 2006; Aron et al. 2007; Mao et al. 2007) and lesion (stroke and tumor) studies (Gossl et al. 2002; Mori et al. 2002; Newton et al. 2006; Schonberg et al. 2006) are also valuable additional forms of validation (Hubbard and Parker 2009). Comparisons with such studies have highlighted the various attributes and pitfalls of different classes of fiber tracking methodologies.

Animal models provide a third avenue for validation. Within this context, the quantity and reliability of tracer data derived from macaque brains, the phylogenetic proximity of macaques and humans, and the ability to acquire data using a comparable imaging protocol make macaques a valuable animal model for validating tractography techniques. Parker et al. (2002) provided the first diffusion-weighted imaging comparison of the macaque and the human brains using fast marching tractography, based on diffusion tensor information. Subsequent comparison work included use of the *q*-ball fiber orientation estimation technique to enable tractography (Tuch et al. 2005), and investigation of macaque brain connectivity patterns (Croxson et al. 2005; Dauguet et al. 2007). However, although these studies showed to some degree the similarity of tractography output with the expected connection information, they did not explore the influence of the range of experimental tractography variables, such as trajectory curvature limits, which restricts the maximum angle through which paths can propagate, and fractional anisotropy (FA) constraints designed to avoid propagation into gray matter. Such variables have been shown (Jones 2010) to lead to variations in the extent and strength of derived pathways. Improvements in MR scanner and computer hardware and processing techniques in the last decade have also allowed the production of higher resolution and signal-to-noise data for MRI tractography, and a resultant need to evaluate the abilities of more recently developed fiber tracking methodologies (Dyrby et al. 2007).

Many invasive tracer studies have characterized the interconnections of the macaque visual system in detail, making it an appropriate model with which to assess the accuracy of tractography outputs (Van Essen et al. 1992). A detailed wiring map of the interconnections in the macaque visual system was first described by Felleman and Van Essen (1991; FVE91). Hence, via comparison with this reference system, diffusion MR images of the macaque brain can be used as a test-bed to validate the output of different tractography approaches between visual cortical regions. Therefore, the aim of this work is to quantify the accuracy of connections identified using MR diffusion-imaging-based tractography in the macaque visual system by comparison with known connections attained from previous invasive tracer studies. We use a probabilistic tractography approach to identify probabilistic connection “strengths” (streamline counts) between visual cortex regions of interest (ROIs) in 2 postmortem macaque brains. Comparisons with the anatomical connections of the FVE91 wiring map allow us to determine the optimum threshold of acceptance of streamline counts, and the accuracy of the tractography method. We assess the effect of distance correction, trajectory curvature and FA constraints on accuracy.

## Materials and Methods

### Image Acquisition

MR high angular resolution diffusion imaging (HARDI) data were acquired in formalin-fixed postmortem brains of 2 rhesus macaques.

#### Dataset 1 (D1)

Imaging data were acquired in a fixed *Macaca mulatta* brain on a 4.7-T Bruker BIOSPEC vertical bore scanner at the Max Planck Institute for Biological Cybernetics, Tübingen, Germany. A surface coil placed over the occipital cortex was used for signal reception. A 2D spin-echo sequence was implemented with time echo (TE) = 78 ms, time repetition (TR) = 9 s, *G*_max_ = 47 mT/m, isotropic voxel resolution 0.8 mm, 61 non-collinear diffusion directions at *b* = 4000 s/mm^2^ (Δ = 39 ms, *δ* = 31 ms), 7 at *b* = 0, number of averages = 4. Total imaging time was approximately 64 h.

#### Dataset 2 (D2)

Imaging data were acquired in a fixed *Macaca fascicularis* brain on a 4.7-T Bruker Avance horizontal bore scanner at the Athinoula A. Martinos Center, Massachusetts General Hospital, Charlestown, MA, USA. Although the brain was subjected to middle cerebral artery occlusion for 1 h, there were no visible ischemic lesions or other pathology on the diffusion tensor images. A 3D spin-echo echo-planar imaging sequence was implemented with 8 shots, TE = 33 ms, TR = 350 ms, *G*_max_ = 380 mT/m, isotropic voxel resolution 0.43 mm, 120 non-collinear diffusion directions at *b* = 8000 s/mm^2^ (Δ = 18.8 ms, *δ* = 6.85 ms), 17 at *b* = 0. Total imaging time was approximately 27 h.

### Image Analysis

#### Data Preprocessing

To improve the signal-to-noise ratio in the diffusion-sensitized images of D1, we applied 5 iterations of 2D anisotropic diffusion smoothing (Perona and Malik 1990; Parker et al. 2000; Pilny and Janacek 2005–2006) using ImageJ (Abramoff et al. 2004).

#### Constrained Spherical Deconvolution and Model-Based Residual Bootstrapping

To perform probabilistic tractography, we first estimate the diffusion probability density function (PDF) describing the likely orientations of axonal fiber bundles within each voxel using constrained spherical deconvolution (CSD; Tournier et al. 2007, 2008) applied to the HARDI data. The single fiber response function, required for the deconvolution process, was obtained from the simulation of a single diffusion tensor with FA of 0.8 and the *b*-factor of the dataset in question. The fiber orientation distribution function was generated with 45 spherical harmonics (*l*_max_ = 8) and was reconstructed at 8000 equidistant points on the sphere, within each voxel. A previously described, model-based bootstrapping (Haroon et al. 2009) was used to generate PDFs to perform probabilistic tractography using the probabilistic index of connectivity (PICo) software package (Parker and Alexander 2003; Parker et al. 2003).

#### Cortical Parcellation

**Figure 1.**
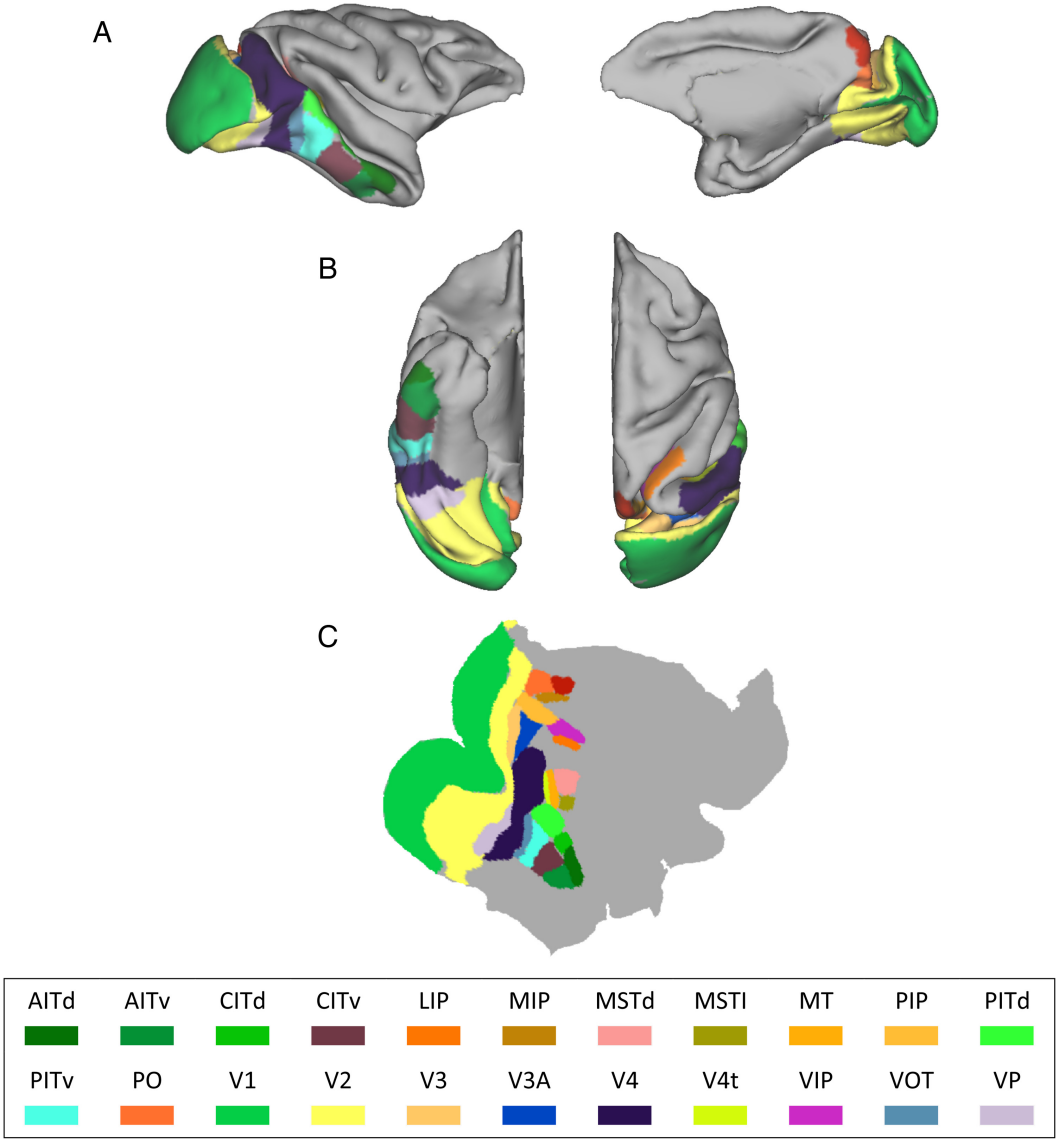
An example of the visual cortex parcellation scheme of Felleman and Van Essen. (*A*) Left: midsagittal view. Right: lateral view. (*B*) Left: ventral–axial view. Right: dorsal–axial view. (*C*) The regions depicted on the cortical flat map of the example macaque brain. AITd and AITv, anterior inferotemporal, dorsal and ventral; CITd and CITv, central inferotemporal, dorsal and ventral; LIP, lateral intraparietal; MIP, medial intraparietal; MSTd and MSTl, medial superior temporal, dorsal and lateral; MT, middle temporal; PIP, posterior intraparietal; PITd and PITv, posterior inferotemporal, dorsal and ventral; PO, parieto-occipital; V1, V2, V3, V4, visual areas 1,2,3,4; V3a, visual area V3a; V4t, V4 transitional; VIP, ventral intraparietal; VOT, ventral occipitotemporal; VP, ventral posterior.

To determine the accuracy of tracking, we first defined the different cortical regions within the visual system on each of the macaque brains. These were then used as seed regions for tracking. We used the cortical partitioning scheme of Felleman and Van Essen (1991; FVE91), available as an MRI volume within the Caret 5.5 software package (Van Essen et al. 2001) for the F99UA1 rhesus macaque brain atlas. We applied nonlinear warping to the anatomical MR brain image volume of F99UA1 to spatially match it to the brain image volumes of our datasets using the Normalize tool (Friston et al. 1995) in SPM5 (Fig. 1). The transformation operations from the nonlinear warping were then applied to the FVE91 cortical partitioning template, bringing the cortical regions into subject space. Given that this segmentation is used to drive the tractography algorithm and that poor matching to the actual gray matter could negatively impact the analysis, the quality of the subject-specific region placements was then manually assessed through a slice-by-slice examination. Wherever required, ROIs were amended and repositioned to correspond to expected cortical landmarks (Saleem and Logothetis 2006), ensuring that they encompassed the gray matter only.

#### Tractography

Twenty-two cortical ROIs were identified in the visual system within both hemispheres of the 2 datasets, allowing us to perform intrahemispheric tracking. As the MRI measurements for D1 were obtained using a surface coil placed over the occipital lobe, we restricted our study to the visual system in both data sets. Each of the visual regions in the spatially matched FVE91 template was used as a seed region for performing probabilistic tractography using PICo (Parker and Alexander 2003; Parker et al. 2003) with 1000 Monte Carlo streamline propagations initiated per voxel of each seed region. For each dataset, a cortico-cortical interconnection matrix was created by measuring how many streamlines from a specified cortical region reached each of the other cortical regions. There was an additional step to impose symmetry: The connections were measured from each A → B pair and from B → A and the maximum number of streamlines from the 2 measurements was taken to be the value of connection. This gave us a symmetrical matrix of “strengths” of cortico-cortical interconnection (SCI) on a scale of 0–100% of initiated streamlines. Connection strengths were determined between all 22 regions within both the left and right hemispheres.

### Comparison with In Vivo Tracing Data

**Figure 2.**
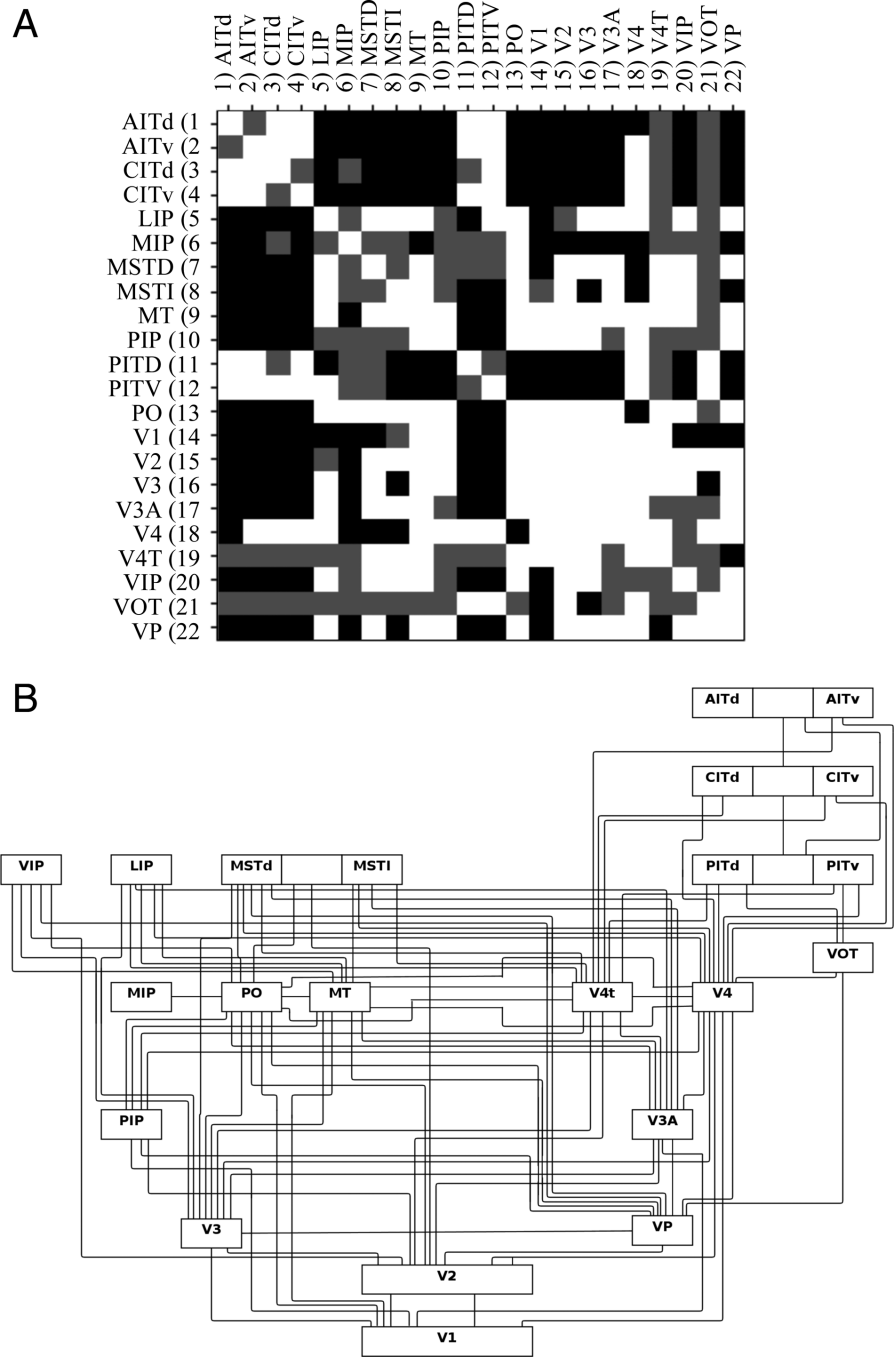
(*A*) The symmetric gold standard connection matrix with regions in an alphabetical order, where white indicates a true connection, black no connection, and gray indeterminate. (*B*) The wiring diagram representations of the in vivo connections from Felleman and Van Essen with labels for different regions as defined in that work.

The SCI matrices were compared with the interconnections described in FVE91, which are based on in vivo tracing results in various species of macaque including *M. mulatta* and *M. fascicularis* (Felleman and Van Essen 1991). True positives (TPs) were the connections established in FVE91 with high confidence (in either the forward or reverse tracing direction) and true negatives (TNs) the connections for which good experimental evidence of no connection existed. Potential connections for which the evidence was deemed unreliable in FVE91 (due to conflicting evidence or lack of findings for that connection) were discounted from our “ground truth” connection matrix (Fig. 2). We use the term “ground truth” not to imply that this matrix definitively represents true connections (as there are limitations to the accuracy of the invasive tracing data), but rather to imply that this matrix was used as a baseline against which the tractography findings were compared.

The accuracy of tractography-derived connections was calculated as the percentage of correctly determined connections (including TP and TN):

(1) $$\text{Accuracy}=\frac{100\times (\text{TP}+\text{TN})}{\text{TP}+\text{TN}+\text{FP}+\text{FN}},$$

where FP is the number of false positive connections and FN false negatives. Accuracy was calculated at every step increase of 1% (between 0 and 100%) in the acceptance threshold value applied to the SCI matrices. If the SCI is above this threshold, then the connection is deemed to be present. Receiver operating characteristic (ROC) curves were also generated by plotting the TP rates against FP rates, where TP_rate_ = TP/(TP + FN) and FP_rate_ = FP/(FP + TN). Finally, % TP was calculated using 100 × TP/(TP + FP) and % TN by 100 × TN/(TN + FN).

Some connections were found to be present in all 4 hemispheres of the macaque tractography data but not present in FVE91, and other connections were present in FVE91 but consistently absent in the tractography data. We thought it possible that these tractography connections that were classified as FPs or FNs may in fact be correct. We considered the accuracy of FVE91 for these connections by comparison with information provided by a further, recently published, quantitative tracer study (Markov et al. 2012), which reports an enhanced description of pathways within the visual system. Despite differences in the partitioning schemes of Felleman and Van Essen and the Markov analysis, we were able to identify analogous regions: visual areas 1,2, visual areas V3a, visual areas 4, V4 transitional, middle temporal, lateral intraparietal, medial intraparietal, ventral intraparietal, and posterior intraparietal were considered to be the same in both schemes, and further regions were identified with different naming conventions: posterior inferotemporal ventral (FVE) = TEO(M), posterior inferotemporal dorsal (FVE) = TEOm(M), central inferotemporal ventral (FVE) = TEpd(M), central inferotemporal dorsal (FVE) = TEpd(M), anterior inferotemporal ventral (FVE) = TEav(M), anterior inferotemporal dorsal (FVE) = TEad(M), PO(FVE) = V6(M), and V6a(M) combined, MST(FVE) = medial superior temporal lateral (M) and MSTd(M) combined, and V3(M) encompassed V3 and ventral posterior in FVE. There was no analogous region in the Markov scheme for the region defined as ventral occipitotemporal in the Felleman and Van Essen scheme.

#### The Effect of Distance Correction, Curvature, and FA Constraints

By recording the average length of the streamlines leaving each seed voxel, the lengths of the connection trajectories originating from each seed region were estimated. As with the SCI matrices, symmetric “length” (in mm) of cortico-cortical interconnection (LCI) matrices were generated for both hemispheres in the 2 datasets; the larger of the lengths measured between 2 regions was used to define the connection in question. The LCI matrices were used to compensate for previously reported (Tomassini et al. 2007; Morris et al. 2008; Jones 2010) distance effects that influence probabilistic tractography results. Two methods of streamline length-based correction were explored. First, as implemented in FSL's probtrackx (Behrens et al. 2007), the values in the SCI matrices were multiplied with the corresponding distance value in the LCI matrices (*R*-correction). Second, SCI matrices were multiplied by the square of the corresponding distance values in the LCI matrix (*R*^2^-correction). To interrogate the success of the corrections, the TP and FP rates were calculated as a function of connection length by dividing all connections into 5 bins, by ordering the connections according to length and placing an equal number of connections in each bin.

We also considered the effects of other constraints and optimizations that are commonly used in tractography experiments. The tractography experiments described above were repeated in both hemispheres for both datasets using 4 different FA streamline propagation termination thresholds of: 0, 0.1, 0.2, and 0.3. These values are based on recommended values that were used in other studies (Kunimatsu et al. 2004; Stadlbauer et al. 2007; Parizel et al. 2007) and are founded upon considerations of the selection of a threshold that distinguishes gray and white matter. As our tracking start and termination regions lie within cortical gray matter, where the FA values may be lower than the thresholds used (i.e., 0.1, 0.2, and 0.3), the use of FA threshold values as streamline propagation termination constraints may end the tracking process before tracking has left the seed mask. To allow for the streamlines to reach the white matter of the brains before the FA termination constraint initiates, a cortical gray matter mask was used to specify regions in which this constraint was not employed. These masks were also used at the far end of the streamlines where the paths penetrate the gray matter. The cortical gray matter mask was generated by combining all the cortical regions derived from the cortical partitioning scheme of FVE91. This was then warped onto the cortex in each dataset.

Another constraint that was explored was the use of curvature thresholds. This constraint is typically employed to allow for the expectation that, in white matter, at a voxel resolution, sharp changes in the direction of fiber pathways are not expected (Schmahmann et al. 2007; Wakana et al. 2007; Behrens and Jbabdi 2009). To test the effects of curvature constraints, curvature-based termination values of 70, 80, 90, and 180° were separately used as constraints at each step of the streamline propagation process.

## Results

### Accuracy of Connections

**Figure 3.**
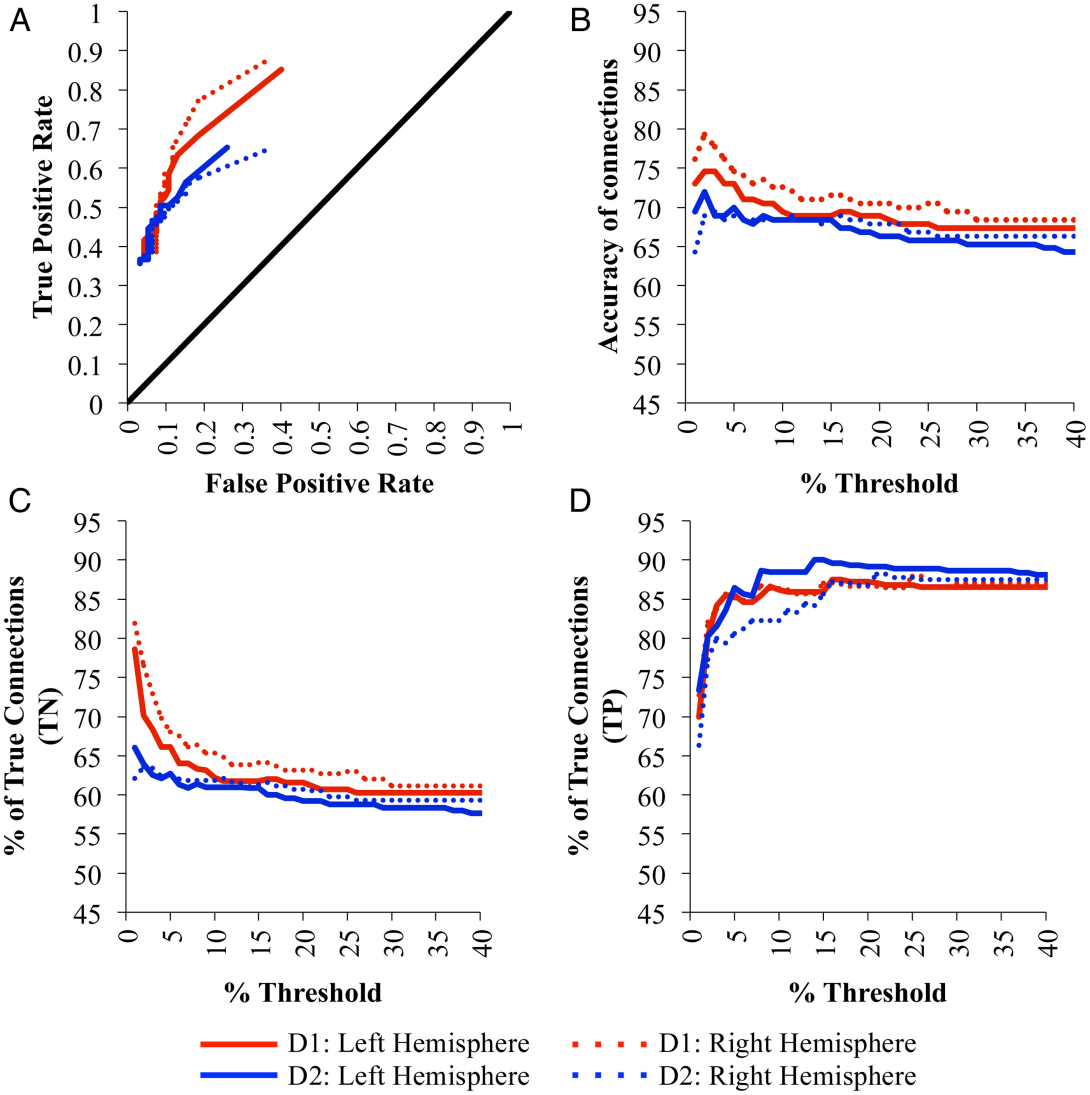
(*A*) The average receiver operating characteristic (ROC) for SCI matrices at a range of acceptance thresholds in comparison with FVE91. (*B*) Accuracy of connections from the SCI matrices at a range of acceptance thresholds from 1 to 40%, compared with FVE91. (*C*) Percentage of TN and (*D*) % of TP connections identified when optimizing the SCI matrices generated for D1 and D2 against the Felleman and Van Essen atlas. Note that results are only shown for acceptance thresholds between 1 and 40%, although the full range of threshold levels up to 100% was tested.

The ROC curves (Fig. 3A) for each hemisphere in each data set show performance that is clearly above chance (black line) for all tested thresholds of SCI. Figure 3B shows the effect of the acceptance threshold for SCI on connection accuracy, which is optimum between 2% and 5% for both datasets. Above this optimum threshold only the strongest connections are accepted, leading to an increase in the percentage of TP connections (Fig. 3D), but a decrease in the percentage of TNs (Fig. 3C) as weaker connections are missed (more FNs). Below the optimum threshold, progressively weaker connections are erroneously accepted (more FPs), reducing the percentage of TPs (Fig. 3D) but increasing the percentage of TNs (Fig. 3C). Average accuracies of 77% and 70% of connections at the optimum thresholds were found in D1 and D2, respectively (Table 1), showing good agreement between the results from each brain, despite quite different acquisition parameters.

**Table 1:** Accuracy of connections

	D1						D2					
	Optimum threshold (%)			Accuracy (%)			Optimum threshold (%)			Accuracy (%)		
	Left	Right	Overall	Left	Right	Overall	Left	Right	Overall	Left	Right	Overall
No correction	2	2	2	75	79	77	2	3	2	72	69	70
*R*-correction	1	1	1	75	77	76	1	1	1	69	69	68
*R*^2^-correction	3	3	3	73	74	74	5	16	5	73	71	71
Curvature ≤70	1	3	1	76	72	74	7	2	6	70	69	70
Curvature ≤80	2	4	2	74	73	73	1	2	1	71	69	69
Curvature ≤90	2	8	2	74	73	73	2	3	3	70	70	70
Curvature ≤180	3	3	3	73	75	74	2	3	2	72	69	70
FA ≥0.0	2	2	2	75	79	77	2	3	2	72	69	70
FA ≥0.1	2	1	1	72	74	72	1	1	1	71	73	71
FA ≥0.2	2	1	1	73	72	72	5	3	3	66	71	68
FA ≥0.3	1	1	1	71	71	71	1	1	1	67	66	68

Note: The results of optimizing D1 and D2 on the Felleman and Van Essen atlas using no distance correction, *R* and *R*^2^ correction; 70, 80, 90, and 180° curvature constraints; and FA ≥0.0, FA ≥0.1, FA ≥0.2, and FA ≥0.3 constraints.

**Figure 4.**
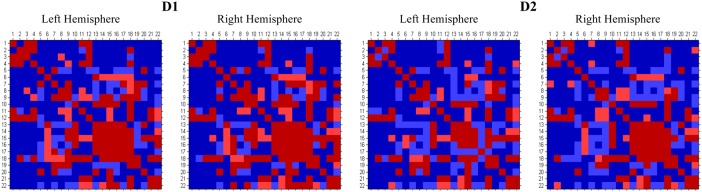
Comparison of the thresholded connection matrices generated for D1 and D2 against the Felleman and Van Essen atlas (Fig. 2A). Regions are provided in an alphabetical order, as defined in Figure 2.

The connection matrices (Fig. 4) show good correspondence between the known connections from in vivo tracing and the diffusion-based connections at the identified optimum acceptance thresholds for each hemisphere. The majority of FN connections were long range (Table 2), involving connections between different lobes. This may be explained by the inherent bias of tractography toward the shortest pathways. FP connections tended to be shorter range, with half being within the same lobe (Table 3). These “false” connections were compared with the results of a more recent quantitative tracer study (Markov et al. 2012) that has identified a number of additional pathways, as indicated by the footnotes “a and b” in Tables 2 and 3. Markov tested 9 of the 18 connections identified as FPs relative to the FVE91 results, and identified 8 previously undocumented true connections and confirmed 1 nonconnection. These newer results are clearly in stronger agreement with the tractography results than the FVE91 results. However, Markov also tested 10 of the 40 FNs (Table 3) and confirmed the existence of 8 connections that were not found with tractography along with 2 connections that were confirmed to be absent.

**Table 2:** Apparent FN connections

	Occipital	Temporal	Parietal
Occipital	**V1–V4t**^a^ **V3–MT**^b^ **V3–V4t** VOT–V3a	V4t–AITd V4t–AITv V4t–CITd^a^	**V3–LIP** **V3a–LIP** **VP–LIP** **V2–MSTi**^b^ **V3a–MSTi** **VP–PIP** **MT–PO**^c^ **V4t–PO** **VP–PO** **VP–VIP** V4t–LIP V4t–MIP V4t–PIP V4t–VIP VOT–MIP VOT–LIP VOT–PIP VOT–VIP VOT–PO
Temporal		**PITv–AITd**^b^	**PITv–LIP**^b^ CITd–MIP^c^
Parietal			**MSTi–LIP** **PO–LIP** **PO–MSTd** **PO–MSTi** **VIP–MSTi** MSTi–MIP PITd–MIP PITv–MIP^a^ PITv–MSTd^b^ PIP–MSTd PIP–MSTi

Connections that are present in the Felleman and Van Essen atlas, but which did not exist in any of the 4 hemispheres of the macaque tractography data. In **bold** are the 20 connections that are clearly identified as present in the Felleman and Van Essen atlas, while there was uncertainty for the 20 connections not highlighted.

^a^Weak connection identified in Markov et al. (2012).

^b^Strong connection identified in Markov et al. (2012).

^c^Connection found to be absent in Markov et al. (2012).

**Table 3:** Apparent FP connections

	Occipital	Temporal	Parietal
Occipital	**V1–VP**^a^	**V2–PITv**^b^ **VP–PITv**^a^ **MT–PITd**^a^ V4t–PITd VOT–CITv	**V2–MIP**^c^ **V3–MIP** **V4–MSTi**^b^
Temporal		AITv–AITd CITv–CITd^a^ PITd–CITd^a^ PITd–PITv^a^	**PITd–MSTi**
Parietal			PIP–LIP PIP–VIP PIP–MIP VIP–MIP

Connections that are present in all 4 hemispheres of the macaque tractography data, but which do not exist in the Felleman and Van Essen atlas. In **bold** are the 8 connections that are clearly identified as not present in the Felleman and Van Essen atlas, while there was uncertainty for the 10 connections not highlighted.

^a^Strong connection identified in Markov et al. (2012).

^b^Weak connection identified in Markov et al. (2012).

^c^Connection found to be absent in Markov et al. (2012).

### The Effects of Distance Correction

**Figure 5.**
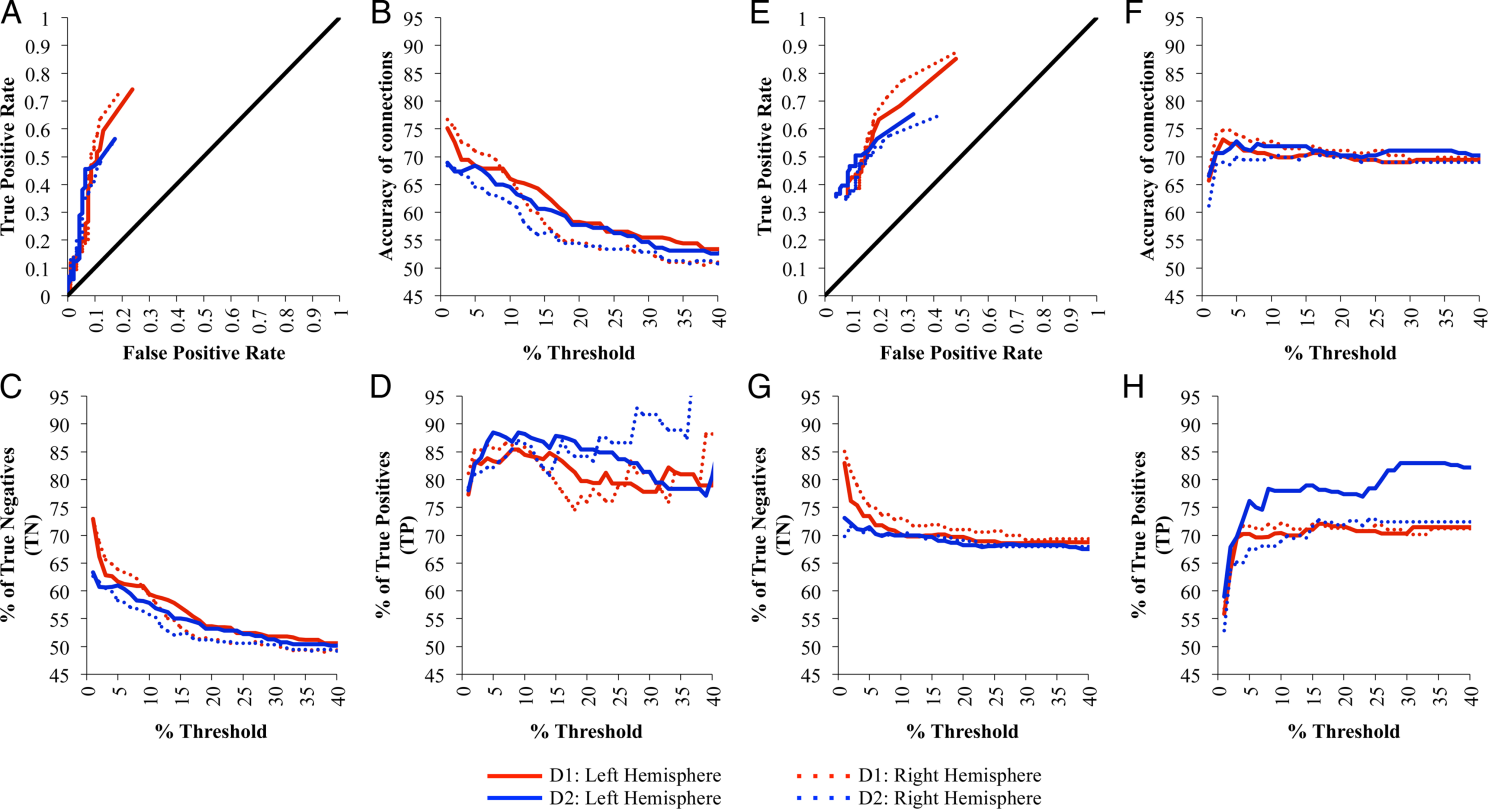
Effect of distance correction, *R*-correction (*A*–*D*) and *R*^2^-correction (*E*–*H*) on: (*A* and *E*) the average ROC for SCI matrices at a range of acceptance thresholds in comparison with FVE91. (*B* and *F*) Accuracy of connections from the SCI matrices at a range of acceptance thresholds from 1% to 40%, compared with FVE91. (*C* and *G*) Percentage of TN and (*D* and *H*) % of TP connections identified when optimizing the SCI matrices generated for D1 and D2 against the Felleman and Van Essen atlas. Note that results are only shown for acceptance thresholds between 1 and 40%, although the full range of threshold levels up to 100% was tested.

**Figure 6.**
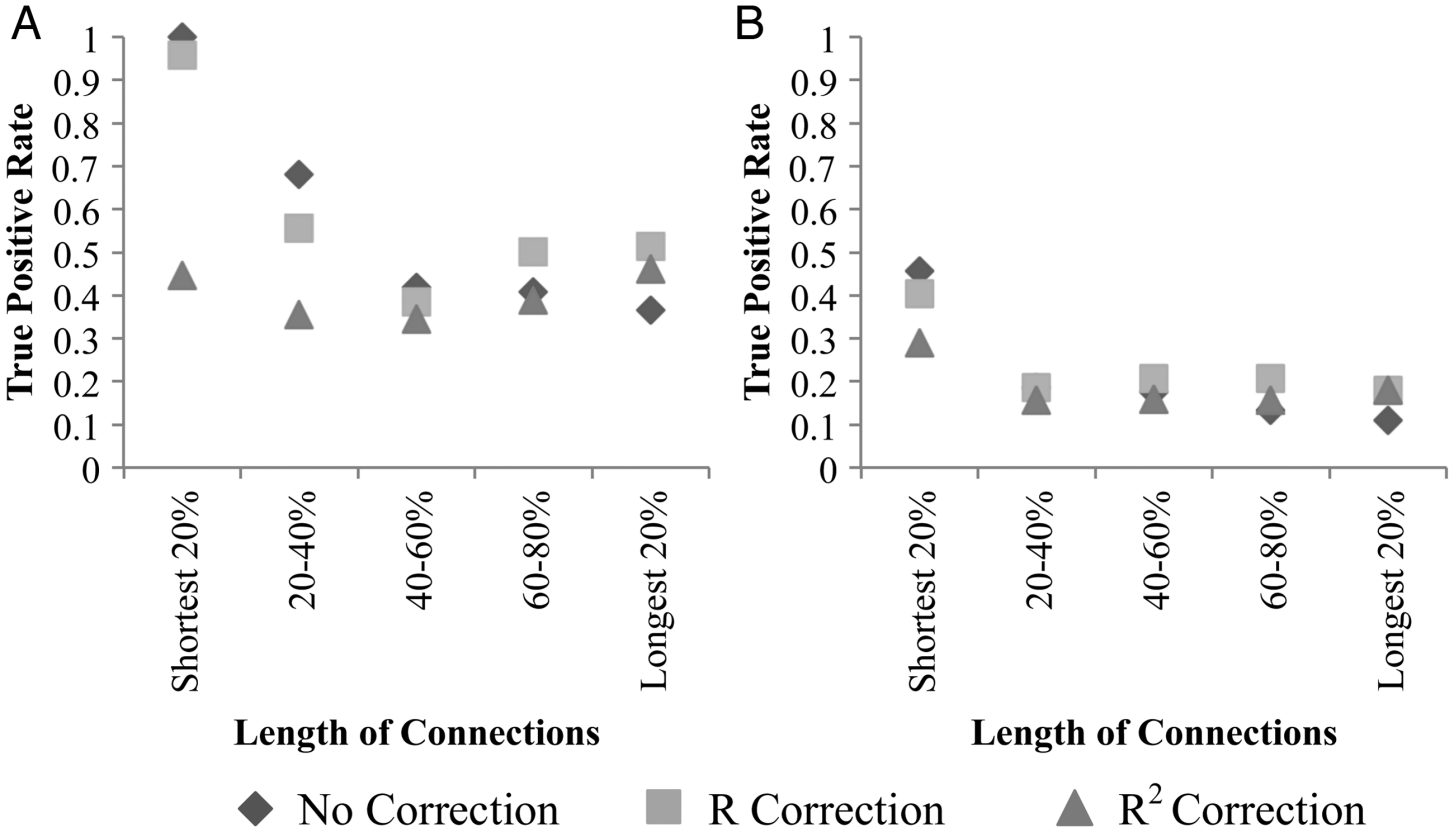
(*A*) TP rates and (*B*) FP rates when using no correction, *R*-correction, and *R*^2^-correction, as a function of distance away from the ROI seed point averaged across D1 and D2.

Figure 5 shows the results of the distance correction methods on accuracy (compare with Fig. 3). Without distance correction, both the TP and FP rates decline with increasing distance away from the ROI seed point (Fig. 6). Both the *R* and *R*^2^-correction^,^ show some improvements in the TP rate identified at long distance (Fig. 6A), but the FP rate also increases (Fig. 6B), resulting in little notable increase in overall accuracy of the identified connections (Table 1).

### The Effects of Streamline Curvature Termination Constraints

Curvature constraints appear to have no clear effect on the accuracy of the results (Table 1). Although the use of a curvature threshold increases the percentage of identified TNs, it also decreases the percentage of identified TPs.

### The Effects of Variations in FA Termination Constraints

The use of higher FA termination thresholds appears to have a negative effect on the accuracy of the tracking results (Table 1), that is, when the FA threshold is increased, the level of accuracy of the tracking results is reduced. These results suggest that, under the experimental conditions used in this work, there is no justification in using any FA threshold to terminate tracking.

## Discussion

Our results demonstrate that a threshold of approximately 2–5% is a good acceptance level for SCI when using probabilistic tracking methods such as PICo. We were able to achieve a tractography-based connection accuracy of 77% in D1 and 70% in D2 relative to the connections that were identified by Felleman and Van Essen. The difference in accuracy found between D1 and D2 is most likely to be due to differences in the data acquisition protocols and the fact that D1 and D2 are from different subspecies of macaque. Considering the uncertainty of false connections in the Felleman and Van Essen work, our results reflect the lower limit of accuracy.

Indeed, further comparison with the results of a recent quantitative tracer study (Markov et al. 2012) supports this conclusion (Tables 2 and 3). Their results suggest that nearly all of the consistent “FP” connections identified by our tractography experiments are true connections, but that the majority of consistent “FNs” are likely to be truly missed connections. This reflects the limited sensitivity of tractography, which is unlikely to be able to reproduce small, fine, or dispersed connection pathways. Furthermore, tractography is thought to be biased toward terminating on gyral crowns rather than on sulcal walls (Jbabdi and Johansen-Berg 2011), which may have contributed to the large number of FNs, which tend to involve small areas in the sulci (Table 2). This suggests that the true accuracy of the tractography results may be greater than suggested by comparison with the Felleman and Van Essen results, and also that a lower threshold of acceptance for the tractography results may be appropriate to capture more of these missed connections. Use of the CoCoMac (http://cocomac.org) database, which is a more up-to-date and comprehensive collection of macaque invasive tracer information, may also offer a better test of accuracy than the Felleman and Van Essen map.

Our analysis did not take into account differences in the physical diameter of the tracts, or the fact that some connections may be associated with subregions within our cortical regions. Both effects are likely to lead to reduced sensitivity of the diffusion MRI tracking methods used in this work; improvements in spatial resolution and in the definition of cortical regions may lead to improvements in the percentage of accurately identified connections and nonconnections. In this study, we did not compensate for variations in the sizes of the cortical regions, that is, more streamlines will propagate from larger cortical regions such as V1 than from smaller regions. While this will reflect the true underlying anatomy, tracer injections tend to be comparable in absolute size and are typically not scaled to area size, so this would introduce some differences between tractography and tracer-derived results.

We investigated the performance of variables such as distance-based corrections, curvature, and FA constraints, which are commonly used when performing tractography, against in vivo tracer results, allowing a better understanding of their true effects. Without distance correction, the TP and FP rates both decline with increasing distance. Both the *R* and *R*^2^-corrections show improvements in the TP rate at long distance, but the FP rate is also increased, resulting in little gain in overall accuracy. Therefore, a more sophisticated correction method is needed if distance effects are to be compensated for.

Unexpectedly, we found that use of the FA thresholds tested reduced the accuracy of the connections found. This is possibly due to the rejection of streamlines that are actually TP pathways. This may be confounded by the necessity to segment gray matter to avoid the application of FA thresholds in these regions. If segmentation is not accurate, or if partial volume problems lead to regions of low FA beyond the identified cortical boundaries, this could contribute to the rejection of TP pathways emerging from the gray matter or entering the gray matter at the terminus of streamlines. In situations where the aim of tractography is not to establish connections between gray matter regions per se, but perhaps to segment a specific white matter pathway without its gray matter entry points, then it may still be possible that FA thresholds could be helpful; our methodology is not able to answer this point definitively.

Although theoretically a curvature threshold appears to be very useful in excluding streamlines that are anatomically doubtful, it may be that such sharp changes in the direction of the streamlines have a minimal effect on the outcome of probabilistic tractography, unlike in deterministic tractography, where a single erroneous change of direction could have severe consequences. This could explain why such a constraint has little or no effect on our results. The slight variation in the results obtained on repeating the experiments using different curvature thresholds may simply be due to the Monte Carlo sampling that is used by PICo, which will introduce some variability. Arguably, the nature of probabilistic tractography dictates that all possible fiber orientations at a given point along a pathway are valid, with the probability of each being chosen determined by the intravoxel PDF alone; additional orientational thresholds are therefore applying post hoc cutoffs on the PDFs, which is at odds with the probabilistic framework of the technique. The lack of influence of curvature constraints in our results may simply be indicating that the bootstrap generation of the PDFs is sufficient to guard against a high probability of pathways with high curvature. It is possible that if our default step size was larger, curvature constraints may have been more important.

Our results are comparable with those of Thomas et al. (2014), showing similar performance on the ROC curves (e.g., compare results in Fig. 3 with the CSD results in Fig. 2 of the work by Thomas) despite consideration of different brain regions. While Thomas interpreted these results in a negative light, we are more optimistic as we believe these measures represent the lower limits of accuracy due to imperfections in the results of the tracer studies. However, we agree that tractography is fundamentally limited in its ability to detect long-range anatomical projections.

## Conclusion

Our results demonstrate that tractography can identify the majority of expected anatomical connections in the visual network of the macaque brain and provide useful data to help define the limitations of the method. However, some caution is needed in interpretation of these results as it is falsely assumed that the invasive tracer studies provide a “gold standard” measure of connections. This limitation may be apparent in our data, where certain connections were present in both MR diffusion imaging datasets, but were absent in the Felleman and Van Essen atlas (a limitation that is partly confirmed by more recent invasive tracking data). Our results therefore represent a lower boundary on the true accuracy of connection identification using tractography. One further limitation of the current study is that it focuses exclusively on identifying the presence or absence of interareal connections, whereas actual connection strengths vary by many orders of magnitude. This comparative approach could prove useful in future studies aiming to test the performance of different tractography algorithms, or to try and identify the optimum acquisition and postprocessing parameters.

## Funding

This work was supported by the UK's BBSRC (grant BB/E002226/1) and the CONNECT consortium, and funded by the future and emerging technologies (FET) program of the EU FP7 framework. Funding to pay the Open Access publication charges for this article was provided by an RCUK grant to the University of Manchester.

## References

[BHU326C1] Abe O Masutani Y Aoki S Yamasue H Yamada H Kasai K Mori H Hayashi N Masumoto T Ohtomo K 2004 Topography of the human corpus callosum using diffusion tensor tractography. J Comput Assist Tomogr. 284:533–539. 15232387 10.1097/00004728-200407000-00016

[BHU326C2] Abramoff MD Magalhaes PJ Ram SJ 2004 Image processing with Image. J Biophotonics Int. 117:36–42.

[BHU326C3] Alexander DC 2005 Multiple-fiber reconstruction algorithms for diffusion MRI. Ann N Y Acad Sci. 10641:113–133. 16394152 10.1196/annals.1340.018

[BHU326C4] Aron AR Behrens TE Smith S Frank MJ Poldrack RA 2007 Triangulating a cognitive control network using diffusion-weighted magnetic resonance imaging (MRI) and functional MRI. J Neurosci. 2714:3743–3752. 17409238 10.1523/JNEUROSCI.0519-07.2007 PMC6672420

[BHU326C5] Basser PJ Mattiello J LeBihan D 1994 MR diffusion tensor spectroscopy and imaging. Biophys J. 661:259–267. 8130344 10.1016/S0006-3495(94)80775-1 PMC1275686

[BHU326C6] Behrens TE Berg HJ Jbabdi S Rushworth MF Woolrich MW 2007 Probabilistic diffusion tractography with multiple fibre orientations: what can we gain? Neuroimage. 341:144–155. 17070705 10.1016/j.neuroimage.2006.09.018 PMC7116582

[BHU326C7] Behrens TEJ Jbabdi S 2009 MR diffusion tractography. In: Johansen-Berg H Behrens TEJ, editors. Diffusion MRI: from quantitative measurement to in-vivo neuroanatomy. London: Academic Press p. 333–351.

[BHU326C9] Campbell JS Siddiqi K Rymar VV Sadikot AF Pike GB 2005 Flow-based fiber tracking with diffusion tensor and q-ball data: validation and comparison to principal diffusion direction techniques. Neuroimage. 274:725–736. 16111897 10.1016/j.neuroimage.2005.05.014

[BHU326C10] Croxson PL Johansen-Berg H Behrens TE Robson MD Pinsk MA Gross CG Richter W Richter MC Kastner S Rushworth MF 2005 Quantitative investigation of connections of the prefrontal cortex in the human and macaque using probabilistic diffusion tractography. J Neurosci. 2539:8854–8866. 16192375 10.1523/JNEUROSCI.1311-05.2005 PMC6725599

[BHU326C11] Dauguet J Peled S Berezovskii V Delzescaux T Warfield SK Born R Westin CF 2007 Comparison of fiber tracts derived from in-vivo DTI tractography with 3D histological neural tract tracer reconstruction on a macaque brain. Neuroimage. 372:530–538. 17604650 10.1016/j.neuroimage.2007.04.067

[BHU326C12] Descoteaux M Savadjiev P Campbell J Pike GB Siddiqi K Deriche R 2007 {Validation and comparison of analytical Q-ball imaging methods}. Washington: IEEE {2007 4TH IEEE International Symposium on Biomedical Imaging: Macro to Nano, Vols 1–3}.

[BHU326C13] Dyrby TB Sogaard LV Parker GJ Alexander DC Lind NM Baare WF Hay-Schmidt A Eriksen N Pakkenberg B Paulson OB 2007 Validation of in vitro probabilistic tractography. Neuroimage. 374:1267–1277. 17706434 10.1016/j.neuroimage.2007.06.022

[BHU326C14] Felleman DJ Van Essen DC 1991 Distributed hierarchical processing in the primate cerebral cortex. Cereb Cortex. 11:1–47. 1822724 10.1093/cercor/1.1.1-a

[BHU326C15] Fieremans E Delputte S Deblaere K Deene YD Truyens B D'Asseler Y Achten E Lemahieu I Walle RVD 2005 A flexible hardware phantom for validation of diffusion imaging sequences. The International Society of Magnetic Resonance Medicine, Miami.

[BHU326C16] Friston KJ Ashburner J Frith CD Poline JB Heather JD Frackowiak RSJ 1995 Spatial registration and normalization of images. Hum Brain Mapp. 33:165–189.

[BHU326C17] Gossl C Fahrmeir L Putz B Auer LM Auer DP 2002 Fiber tracking from DTI using linear state space models: detectability of the pyramidal tract. Neuroimage. 162:378–388. 12030823 10.1006/nimg.2002.1055

[BHU326C18] Guye M Parker GJ Symms M Boulby P Wheeler-Kingshott CA Salek-Haddadi A Barker GJ Duncan JS 2003 Combined functional MRI and tractography to demonstrate the connectivity of the human primary motor cortex in vivo. Neuroimage. 194:1349–1360. 12948693 10.1016/s1053-8119(03)00165-4

[BHU326C19] Haroon HA Morris DM Embleton KV Parker GJM 2009 Model-based residual bootstrap of constrained spherical deconvolution for probabilistic segmentation and tractography. International Society for Magnetic Resonance in Medicine, Honolulu, Hawai'i.

[BHU326C21] Hubbard PL Parker GJM 2009 Validation of tractography. In: Johansen-Berg H Behrens TEJ, editors. Diffusion MRI: from quantitative measurement to in-vivo neuroanatomy. London: Academic Press p. 353–375.

[BHU326C22] Hubbard PL Zhou FL Eichhorn SJ Parker GJM 2015 Biomimetic phantom for the validation of diffusion magnetic resonance imaging. Magn Reson Med. 73:299–305. 24469863 10.1002/mrm.25107

[BHU326C23] Iturria-Medina Y Canales-Rodríguez EJ Melie-García L Valdés-Hernández PA Martínez-Montes E Alemán-Gómez Y Sánchez-Bornot JM 2007 Characterizing brain anatomical connections using diffusion weighted MRI and graph theory. NeuroImage. 363:645–660. 17466539 10.1016/j.neuroimage.2007.02.012

[BHU326C24] Jbabdi S Johansen-Berg H 2011 Tractography—where do we go from here? Brain Connect. 1:169–183. 22433046 10.1089/brain.2011.0033 PMC3677805

[BHU326C25] Johansen-Berg H Rushworth M 2009 Using diffusion imaging to study human connectional anatomy. Ann Rev Neurosci. 321:75–94. 19400718 10.1146/annurev.neuro.051508.135735

[BHU326C26] Jones DK 2010 Challenges and limitations of quantifying brain connectivity in vivo with diffusion MRI. Imaging Med. 23:341–355.

[BHU326C27] Kunimatsu A Aoki S Masutani Y Abe O Hayashi N Mori H Masumoto T Ohtomo K 2004 The optimal trackability threshold of fractional anisotropy for diffusion tensor tractography of the corticospinal tract. Magn Reson Med Sci. 31:11–17. 16093615 10.2463/mrms.3.11

[BHU326C28] Lazar M Alexander AL 2003 An error analysis of white matter tractography methods: synthetic diffusion tensor field simulations. Neuroimage. 202:1140–1153. 14568483 10.1016/S1053-8119(03)00277-5

[BHU326C29] Leemans A Sijbers J Verhoye M Van der Linden A Van Dyck D 2005 Mathematical framework for simulating diffusion tensor MR neural fiber bundles. Magn Reson Med. 534:944–953. 15799061 10.1002/mrm.20418

[BHU326C30] Lin CP Wedeen VJ Chen JH Yao C Tseng WY 2003 Validation of diffusion spectrum magnetic resonance imaging with manganese-enhanced rat optic tracts and ex vivo phantoms. Neuroimage. 193:482–495. 12880782 10.1016/s1053-8119(03)00154-x

[BHU326C31] Mao H Polensek SH Goldstein FC Holder CA Ni C 2007 Diffusion tensor and functional magnetic resonance imaging of diffuse axonal injury and resulting language impairment. J Neuroimaging. 174:292–294. 17894615 10.1111/j.1552-6569.2007.00146.x

[BHU326C32] Markov NT Ercsey-Ravasz MM Ribeiro Gomes AR Lamy C Magrou L Vezoli J Misery P Falchier A Quilodran R Gariel MA 2012 A weighted and directed interareal connectivity matrix for macaque cerebral cortex. Cereb Cortex. 24:17–36. 23010748 10.1093/cercor/bhs270 PMC3862262

[BHU326C33] Mori S Frederiksen K van Zijl PC Stieltjes B Kraut MA Solaiyappan M Pomper MG 2002 Brain white matter anatomy of tumor patients evaluated with diffusion tensor imaging. Ann Neurol. 513:377–380. 11891834 10.1002/ana.10137

[BHU326C34] Morris DM Embleton KV Parker GJM 2008 Probabilistic fibre tracking: differentiation of connections from chance events. NeuroImage. 424:1329–1339. 18619548 10.1016/j.neuroimage.2008.06.012

[BHU326C35] Newton JM Ward NS Parker GJ Deichmann R Alexander DC Friston KJ Frackowiak RS 2006 Non-invasive mapping of corticofugal fibres from multiple motor areas—relevance to stroke recovery. Brain. 129 (Pt 7):1844–1858. 16702192 10.1093/brain/awl106 PMC3718077

[BHU326C36] Parizel PM Rompaey VV Loock N Hecke WV Goethem JWV Leemans A Sijbers J 2007 Influence of user-defined parameters on diffusion tensor tractography of the corticospinal tract. Neuroradiology J. 20:139–147. 10.1177/197140090702000202 24299634

[BHU326C37] Parker GJ Alexander DC 2003 Probabilistic Monte Carlo based mapping of cerebral connections utilising whole-brain crossing fibre information. Inf Process Med Imaging. 18:684–695. 15344498 10.1007/978-3-540-45087-0_57

[BHU326C38] Parker GJ Haroon HA Wheeler-Kingshott CA 2003 A framework for a streamline-based probabilistic index of connectivity (PICo) using a structural interpretation of MRI diffusion measurements. J Magn Reson Imaging. 182:242–254. 12884338 10.1002/jmri.10350

[BHU326C39] Parker GJ Stephan KE Barker GJ Rowe JB MacManus DG Wheeler-Kingshott CA Ciccarelli O Passingham RE Spinks RL Lemon RN 2002 Initial demonstration of in vivo tracing of axonal projections in the macaque brain and comparison with the human brain using diffusion tensor imaging and fast marching tractography. Neuroimage. 154:797–809. 11906221 10.1006/nimg.2001.0994

[BHU326C40] Parker GJM Baustert I Tanner SF Leach MO 2000 Improving image quality and T1 measurements using saturation recovery turboFLASH with an approximate K-space normalisation filter. Magn Reson Imaging. 182:157–167. 10722976 10.1016/s0730-725x(99)00124-1

[BHU326C41] Perona P Malik J 1990 Scale-space and edge detection using anisotropic diffusion. IEEE Trans Pattern Anal Mach Intell. 127:629–639.

[BHU326C42] Perrin M Poupon C Rieul B Leroux P Constantinesco A Mangin JF Lebihan D 2005 Validation of q-ball imaging with a diffusion fibre-crossing phantom on a clinical scanner. Philos Trans R Soc Lond B Biol Sci. 3601457:881–891. 16087433 10.1098/rstb.2005.1650 PMC1854933

[BHU326C43] Pilny V Janacek J 2005–2006 “Anisotropic Diffusion 2D.” http://rsb.info.nih.gov/ij/plugins/anisotropic-diffusion-2d.html.

[BHU326C44] Powell HW Parker GJ Alexander DC Symms MR Boulby PA Wheeler-Kingshott CA Barker GJ Noppeney U Koepp MJ Duncan JS 2006 Hemispheric asymmetries in language-related pathways: a combined functional MRI and tractography study. Neuroimage. 321:388–399. 16632380 10.1016/j.neuroimage.2006.03.011

[BHU326C45] Sakaie KE Lowe MJ 2007 An objective method for regularization of fiber orientation distributions derived from diffusion-weighted MRI. NeuroImage. 341:169–176. 17030125 10.1016/j.neuroimage.2006.08.034

[BHU326C46] Saleem KS Logothetis NK 2006 A combined MRI and histology atlas of the rhesus monkey brain in stereotaxic coordinates. London: Academic Press.

[BHU326C47] Savadjiev P Campbell JS Pike GB Siddiqi K 2006 3D curve inference for diffusion MRI regularization and fibre tractography. Med Image Anal. 105:799–813. 16919994 10.1016/j.media.2006.06.009

[BHU326C48] Schmahmann JD Pandya DN Wang R Dai G D'Arceuil HE de Crespigny AJ Wedeen VJ 2007 Association fibre pathways of the brain: parallel observations from diffusion spectrum imaging and autoradiography. Brain. 130 (Pt 3):630–653. 17293361 10.1093/brain/awl359

[BHU326C49] Schonberg T Pianka P Hendler T Pasternak O Assaf Y 2006 Characterization of displaced white matter by brain tumors using combined DTI and fMRI. Neuroimage. 304:1100–1111. 16427322 10.1016/j.neuroimage.2005.11.015

[BHU326C50] Stadlbauer A Nimsky C Buslei R Salomonowitz E Hammen T Buchfelder M Moser E Ernst-Stecken A Ganslandt O 2007 Diffusion tensor imaging and optimized fiber tracking in glioma patients: histopathologic evaluation of tumor-invaded white matter structures. NeuroImage. 343:949–956. 17166744 10.1016/j.neuroimage.2006.08.051

[BHU326C51] Thomas C Ye FQ Irfanoglu MO Modi P Saleem KS Leopold DA Pierpaoli C 2014 Anatomical accuracy of brain connections derived from diffusion MRI tractrography is inherently limited. Proc Natl Acad Sci USA. 111 (46):16574–16579. 25368179 10.1073/pnas.1405672111 PMC4246325

[BHU326C52] Tomassini V Jbabdi S Klein JC Behrens TE Pozzilli C Matthews PM Rushworth MF Johansen-Berg H 2007 Diffusion-weighted imaging tractography-based parcellation of the human lateral premotor cortex identifies dorsal and ventral subregions with anatomical and functional specializations. J Neurosci. 2738:10259–10269. 17881532 10.1523/JNEUROSCI.2144-07.2007 PMC6672665

[BHU326C53] Toosy AT Ciccarelli O Parker GJ Wheeler-Kingshott CA Miller DH Thompson AJ 2004 Characterizing function-structure relationships in the human visual system with functional MRI and diffusion tensor imaging. Neuroimage. 214:1452–1463. 15050570 10.1016/j.neuroimage.2003.11.022

[BHU326C54] Tournier JD Calamante F Connelly A 2007 Robust determination of the fibre orientation distribution in diffusion MRI: non-negativity constrained super-resolved spherical deconvolution. Neuroimage. 354:1459–1472. 17379540 10.1016/j.neuroimage.2007.02.016

[BHU326C55] Tournier JD Calamante F King MD Gadian DG Connelly A 2002 Limitations and requirements of diffusion tensor fiber tracking: an assessment using simulations. Magn Reson Med. 474:701–708. 11948731 10.1002/mrm.10116

[BHU326C56] Tournier JD Yeh CH Calamante F Cho KH Connelly A Lin CP 2008 Resolving crossing fibres using constrained spherical deconvolution: validation using diffusion-weighted imaging phantom data. Neuroimage. 422:617–625. 18583153 10.1016/j.neuroimage.2008.05.002

[BHU326C57] Tuch DS Wisco JJ Khachaturian MH Ekstrom LB Kotter R Vanduffel W 2005 Q-ball imaging of macaque white matter architecture. Philos Trans R Soc Lond B Biol Sci. 3601457:869–879. 16087432 10.1098/rstb.2005.1651 PMC1854934

[BHU326C58] Van Donkelaar CC Kretzers LJ Bovendeerd PH Lataster LM Nicolay K Janssen JD Drost MR 1999 Diffusion tensor imaging in biomechanical studies of skeletal muscle function. J Anat. 194 (Pt 1):79–88. 10227669 10.1046/j.1469-7580.1999.19410079.x PMC1467896

[BHU326C59] van Doorn A Bovendeerd PH Nicolay K Drost MR Janssen JD 1996 Determination of muscle fibre orientation using diffusion-weighted MRI. Eur J Morphol. 341:5–10. 8743092 10.1076/ejom.34.1.5.13156

[BHU326C60] Van Essen DC Anderson CH Felleman DJ 1992 Information processing in the primate visual system: an integrated systems perspective. Science. 2555043:419–423. 1734518 10.1126/science.1734518

[BHU326C61] Van Essen DC Drury HA Dickson J Harwell J Hanlon D Anderson CH 2001 An integrated software suite for surface-based analyses of cerebral cortex. J Am Med Inform Assoc. 85:443–459. 11522765 10.1136/jamia.2001.0080443 PMC131042

[BHU326C62] von dem Hagen EA Henkelman RM 2002 Orientational diffusion reflects fiber structure within a voxel. Magn Reson Med. 483:454–459. 12210909 10.1002/mrm.10250

[BHU326C63] Wakana S Caprihan A Panzenboeck MM Fallon JH Perry M Gollub RL Hua K Zhang J Jiang H Dubey P 2007 Reproducibility of quantitative tractography methods applied to cerebral white matter. NeuroImage. 363:630–644. 17481925 10.1016/j.neuroimage.2007.02.049 PMC2350213

[BHU326C64] Watanabe M Aoki S Masutani Y Abe O Hayashi N Masumoto T Mori H Kabasawa H Ohtomo K 2006 Flexible ex vivo phantoms for validation of diffusion tensor tractography on a clinical scanner. Radiat Med. 249:605–609. 17111268 10.1007/s11604-006-0076-4

[BHU326C66] Yanasak N Allison J 2006 Use of capillaries in the construction of an MRI phantom for the assessment of diffusion tensor imaging: demonstration of performance. Magn Reson Imaging. 2410:1349–1361. 17145407 10.1016/j.mri.2006.08.001

